# Typhus and Typhoid Fever

**Published:** 1863-02

**Authors:** 


*Typhus and Typhoid Fever.*—The number of those who believe in the specific or essential diversity of typhus and typhoid fever is constantly lessening. They are the result of the same poison co-operating in influence with other causes acting upon individuals. One person, whose various excretory organs are in perfect order, resists altogether the pernicious effects of the poison, especially if exposed but a limited time to its operation. Another partially or imperfectly, the result being slight febrile or constitutional disturbance, or perchance, manifesting the symptoms of the slow, nervous fever of Armstrong.

Another still, whose intestinal excretories, particularly the patches of Peyer, are called into energetic operation by its local influence, will exhibit common typhoid fever—the enteric fever of Wood. And another, eliminating little or none, illustrates the potent impression of the typh *materies morbi* upon the brain and nervous system, or typhus. Or, again, it may ultimate in local lesion of particular organs, as of the lungs, in which case the local affection may overshadow all other manifestations. Or the long continued operation of the same causes may determine the closely allied, if not identical, disease, tuberculous deposit.

Diseases are not entities—least of all are typhus and typhoid. The material change which constitutes disease is the product of all the causes, whether internal or external, conjointly acting upon single organs or the whole system.

The therapeutics of the typh diseases from the mildest to the gravest, from the most acute to the slowest chronic, will be found reducible to the same general principles. Pulmonary phthisis and typhoid fever have a common parentage—concurrent causes determine the result.

There are no specifics for either. Expectorants in the one, or antiphlogistics in the other, though they may respectively prove occasionally useful, are, so to speak, only accidentally so. We must refer to the causes in each individual case, and either remove them, or remove the patient from the sphere of their influence. Without this, medication, whether by antiphlogistics, or stimulants, or alteratives, is, at the best, but null.

Preventing further access of poison, we are to favor elimination and necessary tissue metamorphosis, according to the special indications. Repressing excessive action of any organ immediately involved. And then restore the proper constitution of the blood by nutriment, choosing simply the most easily digestible and that which is physiologically necessary. Whether tonics or stimulants are necessary depends wholly upon their favoring and energizing nutrition—a point which observation must determine in each individual. Alcohol may be required freely, or moderately, or not at all. "Starving a fever," and putting the tuberculous man on an exclusively farinaceous diet, are barbarisms now gone into desuetude.

The appetite is not a test of the want. The same poison, which in its most noticeable result engenders delirium or coma, in its first effect dulls the nerves responsive to the constitutional want. The man who feels cold is not freezing, but he who sleepily denies any sensation of cold whatever. Feeding the typhoid patient is one of the best methods of awakening the appetite for food. Mercurials, or even Terebinthinates, are among the worst.

All medication should be considered as merely subsidiary to the digestive and nutrient processes.

Dr. John Hjaltelin, of Iceland, says that the indications are:

1st. To prevent overcrowding in the farm huts and cabins as far as possible.

2d. To provide perfect ventillation by opening the windows, or making holes for the outlet of the vitiated air.

3d. To destroy every offensive odor about the sick, and even the smell of the sickness itself.

4th. To introduce cleanliness in every respect.

5th. To clean the bowels of the patient as soon as possible in an effective and perfect manner.

6th. To destroy instantly the odor of evacuations.

7th. To use disinfecting medicines internally in a bold and consequential manner.

8th. To support the strength of the patient by easily digestible but nourishing food.

Which rules are applicable the world over, as well as in Iceland.

Dr. Hjaltelin, naively but most sensibly, remarks:

"It seems to me that many physicians are too much afraid of using nourishing diet in typhoid fever, forgetting the great loss of nitrogenous compounds which this sickness, by the large excretion of urea, produces. I have seen many typhus patients in this country, who, as soon as they were able, took very nourishing food, which would never be allowed in the hospitals of Europe, recover speedily; and comparing this fact with the languishing and protracted recovery in the hospitals, I conclude that nourishing food in the latter stages of this fever is quite indispensable."

Commence earlier with your "nourishing food," good Dr. Hjaltelin, and your heart shall be still more rejoiced at the result!

